# Immunocontraception in Wild Horses (*Equus caballus*) Extends Reproductive Cycling Beyond the Normal Breeding Season

**DOI:** 10.1371/journal.pone.0013635

**Published:** 2010-10-26

**Authors:** Cassandra M. V. Nuñez, James S. Adelman, Daniel I. Rubenstein

**Affiliations:** Department of Ecology and Evolutionary Biology, Princeton University, Princeton, New Jersey, United States of America; Institut Pluridisciplinaire Hubert Curien, France

## Abstract

**Background:**

Although the physiological effects of immunocontraceptive treatment with porcine zona pellucida (PZP) have been well studied, little is known about PZP's effects on the scheduling of reproductive cycling. Recent behavioral research has suggested that recipients of PZP extend the receptive breeding period into what is normally the non-breeding season.

**Methodology/Principal Findings:**

To determine if this is the case, we compiled foaling data from wild horses (*Equus caballus*) living on Shackleford Banks, North Carolina for 4 years pre- and 8 years post-contraception management with PZP (pre-contraception, n = 65 births from 45 mares; post-contraception, n = 97 births from 46 mares). Gestation lasts approximately 11–12 months in wild horses, placing conception at approximately 11.5 months prior to birth. Since the contraception program began in January 2000, foaling has occurred over a significantly broader range than it had before the contraception program. Foaling in PZP recipients (n = 45 births from 27 mares) has consistently occurred over a broader range than has foaling in non-recipients (n = 52 births from 19 mares). In addition, current recipients of PZP foaled later in the year than did prior recipient and non-recipient mares. Females receiving more consecutive PZP applications gave birth later in the season than did females receiving fewer applications. Finally, the efficacy of PZP declined with increasing consecutive applications before reaching 100% after five consecutive applications.

**Conclusions/Significance:**

For a gregarious species such as the horse, the extension of reproductive cycling into the fall months has important social consequences, including decreased group stability and the extension of male reproductive behavior. In addition, reproductive cycling into the fall months could have long-term effects on foal survivorship. Managers should consider these factors before enacting immunocontraceptive programs in new populations. We suggest minor alterations to management strategies to help alleviate such unintended effects in new populations.

## Introduction

The extirpation of predator species has resulted in the expansion of free-ranging ungulate populations in North America [1], necessitating their regulation. Immunocontraceptive management has become increasingly popular as culling programs are seldom well-received by the general public. In females, the most common form of immunocontraception, porcine zona pellucida (PZP), stimulates the production of antibodies that bind sperm receptors on the egg's surface, thereby preventing sperm attachment and fertilization [2].

In recent decades, the wild horse (*Equus caballus*) has become a model for evaluating the effects of PZP, thanks chiefly to the seminal nine-year study of contracepted mares on Assateague Island National Seashore [3]. Subsequent research on this population has shown that PZP has little to no effect on recipient physiology or behavior [4], [5], [6]. For example, in Assateague horses, PZP has no effect on the duration of individual estrous cycles [6], and researchers have reported only minor ovulation failure and depressed urinary oestrogen concentrations with repeated applications [4]. However, behavioral research on the horses of Shackleford Banks, North Carolina, and other wild ungulate species suggests that PZP affects the reproductive physiology of recipient animals [7], [8], [9]. In each of these studies, females treated with PZP extended reproductive behaviors into the non-breeding season. While these results are consistent with an extension of ovulatory cycling into the post-breeding season when most females are normally anovulatory [10], [11], this possibility has not yet been tested.

Mares are seasonally polyestrous and extended estrous periods have been documented [11]. Tropical species, for example, are less strictly seasonal, and in some cases, reproduce throughout the year [12], [13]. In addition, variability in the cycling schedules and receptivity of individual mares [14], and the performance of estrous behavior and copulatory activities during the non-breeding season [15] have been documented in temperate species. Such variation in ovulatory scheduling and receptivity suggest that the seasonality of reproductive behavior in *Equus caballus* females is characterized by a substantial degree of plasticity. As the physiological state of contracepted animals has been significantly altered, the possibility of prolonged extended cycling is even more feasible.

In wild horse societies, the harem is the core social group, consisting of usually one, but sometimes two or three harem male(s), one to several female(s), and their offspring [16], [17], [18], [19], [20], [21]. Harem males will sometimes fight to acquire mares from other groups, but stallions almost always retain their mares [18], [19], [20], [22]. In temperate environments, food availability is lower during the fall and winter months and free-ranging horses will alter their activity to maximize food intake and reduce energetic costs [23], [24]. Mares are typically anovulatory at this time and sexual behavior in males is largely absent [11], [14].

On Shackleford Banks, increased reproductive behavior in the post-breeding season by mares has resulted in increased male attentiveness [9]. Such behavior (by males) has been shown to restrict the movement of females, thus reducing their grazing efficiency [21], [25]. The occurrence of this behavior during a time of year when animals typically increase group spread to acquire adequate forage [19], [24], represents a change in behavior fundamental to the animals' survival [26], [27]. Offspring conceived during the post-breeding season are likely subject to decreased resource availability as lower quality forage can affect mares' ability to produce sufficient milk [27]. Finally, regardless of the timing of titer decline post-treatment [9], [28], [58], when anti-PZP antibody titers decrease during the fall months [28], [29], extended reproductive cycling among recipient mares will increase their chances of conception, thereby lowering the vaccine's overall efficacy. Determining whether PZP recipients are likely to extend reproductive cycling is therefore of great importance if managers are to limit animal numbers while still maintaining functional, healthy populations.

Here we use the birth dates of foals to estimate dates of conception for PZP recipient and non-recipient mares on Shackleford Banks, North Carolina. Gestation length in wild horses is 11–12 months [30]. Therefore, dates of conception can be reliably estimated as approximately 11.5 months prior to birth. Breeding normally occurs from March through August, with most births occurring in April and May [11]. Given the extension of reproductive behavior in PZP- treated mares [9], we hypothesize that PZP recipients will extend cycling into the non-breeding season more often than will non-recipients. Therefore, when PZP recipients conceive and give birth, they will do so later on average and over a wider range of months than will non-recipients.

## Methods

### Study area

This study was conducted on Shackleford Banks, a barrier island located approximately 3 km off the coast of North Carolina, USA. The island was 15 km in length, and varied between 0.5 and 3 km in width. The horse population on Shackleford Banks has been co-managed by the National Park Service and the Foundation for Shackleford Horses since 1996.

### Study subjects

The reproductive units of Shackleford horses are typical of feral equids. They are coherent harem groups of one, or sometimes two or three stallion(s), one to several mare(s) and their offspring [18]. Predominantly, the harem groups are not territorial and animals move within overlapping home ranges, although this has not always been the case [18].

### Management

#### PZP Contraception

In January 2000, the National Park Service began the application of PZP for the purposes of immunocontraception. The National Park Service administers PZP in the spring (late February through April) each year. Mares receive their initial treatment at 1.5–2 years of age. Each injection includes 100 micrograms of PZP with an adjuvant (combined at the darting site). Initial doses include Freund's Complete Adjuvant, Modified, Mycobacterium butyricum (Calbiochem #344289). All succeeding doses include Freund's Incomplete Adjuvant (Sigma #F5506). In a given year, an average of 63% of all reproductive mares are inoculated with the vaccine (range = 37–88%). The authors of the present study are not and have never been in charge of making management decisions regarding this herd.

#### Gathers and Removals

The National Park Service began their management of the Shackleford Banks population prior to the use of PZP, performing five gathers between November 1996 and January 2000. During these gathers the majority of the population was rounded up and individuals testing positive for equine infectious anemia were removed and either euthanized or quarantined. As such forms of management could conceivably influence reproductive cycling, we analyzed foaling dates before and after gathers (but before contraception management) using a linear mixed effects model (see Statistical Analyses).

From January 2000 – January 2008, 38 foals (conceived due to contraception failure or administration scheduling) were removed from the island for the purpose of population control. The majority of removals (92%) were conducted in the January following the foals' birth.

### Foaling Data

We recorded foaling data before contraception management (1995–1997) during a study of mother-infant behavior [31]. We obtained foaling data for 2000 and post-contraception years (2001–2008) from the National Park Service at Cape Lookout National Seashore, North Carolina. Although PZP was first administered in 2000, foals born in this year were conceived before contraception management. As such, we considered 2000 a pre-contraception year.

We identified individual horses by color, sex, age, physical condition, and other distinguishing markings including freeze brands. We monitored pregnant mares 2–3 times per week to ensure accurate estimation of foaling date. Births are not commonly witnessed, as mares will generally give birth away from their harem group [31]. This does not significantly affect the determination of birth dates because typically, mares return to their groups within hours (depending on the length of the foaling process and the mares' and foals' condition afterward). In the event that we did not locate mares for more than one week, the birth dates of new foals were estimated by comparing the condition of the foals' coat, mane and tail hair, and locomotor ability to that of foals for which the exact age was known. Using these methods, we were able to estimate birthdays within a range of 1–2 days to 1 week. Focal animals in this study were observed from a minimum of 15 m away at all times. The observers did not obstruct or manipulate the animals' natural behavior in any way. As such, approval for this study by a review board or ethics committee was unnecessary.

We designated births as coming from current recipient, prior recipient, or non-recipient mares. Current recipients received PZP treatment the year they conceived (the year previous to foaling). Prior recipients received PZP treatment at some point earlier in their lifetime, but not the year of conception. Non-recipients had never received PZP at the time of conception.

### Physical Condition

Physical condition is an important factor to consider when monitoring reproductive behaviors. Animals in better condition will have more resources to allocate to mating behavior and physiology than will animals in poorer condition. We assessed mare condition via rump scoring. We determined rump scores examining the curvature of the line between the tailbone and the point of the hip. Scores were based on a scale from 1 to 5; a score of 1 being the poorest [32].

### Weather Data

We collected all climatology data from the Morehead City WNW Station at 34° 44′N; 76° 44′W, approximately 8 km from the study site. We obtained all data from the National Climate Data Center (2009) [33].

### Statistical Analyses

We analyzed data in R version 2.11.1 (R Core Group, Vienna Austria) and JMP, version 7 (SAS, Cary NC, USA). We used Fligner-Killeen tests to determine if variation in foaling dates differed before and after contraception management and between PZP recipients and non-recipients. This test is more conservative than Fisher's or Bartlett's tests and is less sensitive to outliers and non-normal data [34]. Results from these tests show Bonferroni corrected *P*-values.

We used linear mixed effects models to analyze monthly weather patterns. These statistical models included year as a random effect and the following fixed effects: linear through fourth order polynomial functions of month (to account for nonlinear fluctuations in temperature and rainfall), management regime (before or after contraception management), and two-way interactions with management regime. We selected best fit models using Akaike's Information Criterion adjusted for small sample size (AICc). Because temperature data were temporally autocorrelated, we included an autoregressive moving average correlation structure [35]. For each year of the study, we calculated the mean of the monthly residual values from both the rainfall and temperature models from July through November. We included these terms in the mixed effects models described below to determine if weather affected foaling date.

To test for differences in foaling date with PZP treatment, while controlling for pseudo-replication and unequal variances, we used a linear mixed effects model. The model included mare ID as a random effect and a function allowing different variances across groups (pre-contraception management, post-contraception management non-recipient, post-contraception management current recipient, and post-contraception management prior recipient) [35]. We included mare age as a covariate, as this has been suggested to influence female reproductive behaviors and physiology [36], [37]. Additionally, a one-way ANOVA revealed no differences in mare age among treatment groups (F_2,84_ = 2.03, *P* = 0.14), suggesting that age did not confound differences among groups. From the above mixed effects model, differences in foaling dates among current, prior, and non-recipients were calculated using an F-test for linear combinations [35].

As gathers could have affected foaling date, we used a linear mixed effects model to test for differences between foaling dates before and after gathers (but before contraception management). After controlling for weather and mare age, mean foaling date did not differ before and after gathers (*P* = 0.63). Additionally, the range of birth dates did not differ significantly between these groups (SD before gathers  = 1.04, SD after gathers  = 0.77, *P* = 0.36). Finally, AICc from our analysis of foaling dates (see Results) suggested that combining data before and after gathers yielded a more parsimonious model than did separating those categories (AICc separating pre- and post-gathers  = 551.02, AICc combining pre- and post-gathers  = 550.60). Consequently these groups were combined as pre-contraception management animals for all analyses.

To determine whether the number of PZP applications (total or consecutive) or the number of years between pregnancies (total or consecutive) influenced foaling date, we ran separate linear mixed effects models using these as predictor variables. Each model used only data from PZP recipients and included mare ID as a random effect. To determine if results from this analysis could be influenced by year, we ran a linear mixed effects model on post-management, non-recipients predicting foaling date by year.

We analyzed PZP efficacy using a generalized linear mixed effects model with a binomial error distribution and mare ID as a random effect [38]. This analyzed the probability of conceiving using age at first PZP application and the number of consecutive PZP applications as fixed effects. All mixed effects models initially included two-way interactions, which were removed if their *P*-values were >0.10.

## Results

### Foaling

**Figure 1:**
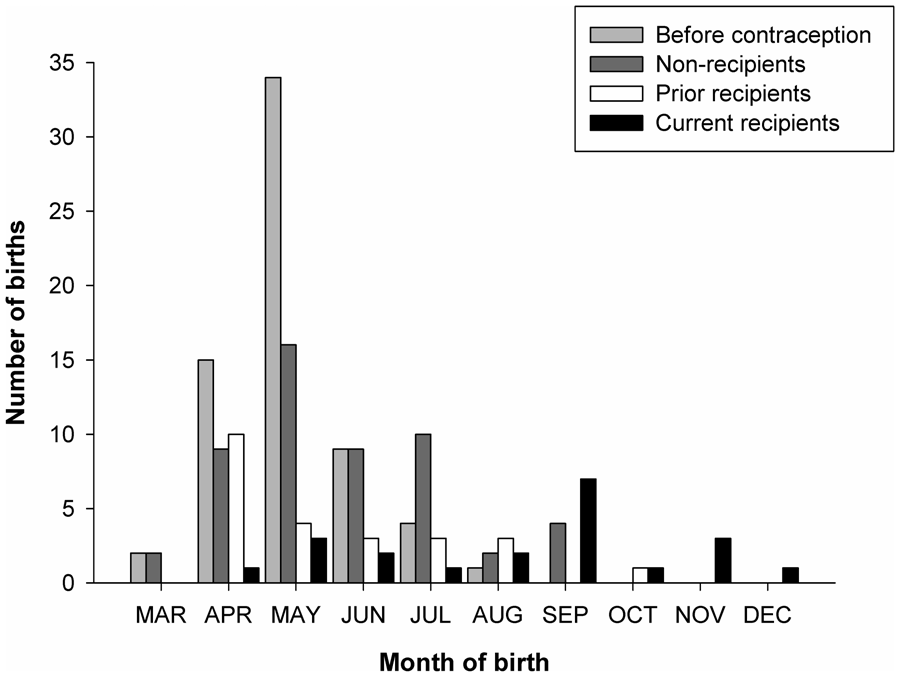
The distribution of births for mares on Shackleford Banks, NC, pre-contraception and post-contraception management. Mares gave birth over a wider range of months after the onset of contraception; this effect was more pronounced in PZP recipients than non-recipients.

After contraception management, foaling occurred over a broader range of months than before contraception management (see Fig. 1, SD pre-contraception  = 0.91 months, SD post-contraception  = 2.04 months; Fligner-Killeen Test for Homogeneity of Variances: Median Chi Square  = 28.10, *P*<0.0001; pre-contraception: n = 65 births from 45 mares over 4 years; post-contraception: n = 97 births from 46 mares, over 8 years). In the eight years following contraception management, mares receiving PZP foaled over a broader range of months than did non-recipient mares (see Fig. 1, SD recipients  = 2.40 months, SD non-recipients  = 1.54 months; Fligner-Killeen Test for Homogeneity of Variances: Median Chi Square  = 11.76, *P* = 0.001; recipients: n = 45 births from 27 mares; non-recipients: n = 52 births from 19 mares).

On average, current PZP recipients gave birth 3.36 months later than did pre-contraception mares, according to our linear mixed effects model, which controlled for heterogeneity of variances, weather influences, and mare age (estimate = 3.36, SE = 0.51, t = 6.64, *P*<0.001; overall model: Likelihood ratio (compared to null model): 40.79, generalized r^2^ = 0.48, *P*<0.001). Mares that had received PZP earlier in their lifetime, but not during the year of conception (prior recipients), gave birth 0.90 months later than pre-contraception mares on average (estimate = 0.90, SE = 0.40, t = 2.27, *P* = 0.03). Non-recipient mares that never received PZP themselves, but gave birth after the general population was managed with PZP, gave birth 1.01 months later than pre-contraception mares on average (estimate = 1.01, SE = 0.25, t = 4.04, *P* = 0.001). This translates into current PZP recipients giving birth 2.34 months later than non-recipient animals and 2.46 months later than prior recipients (F-tests for linear combinations: F>16, *P*<0.001 for each comparison). Furthermore, birth dates were about 0.38 months later for each degree centigrade above average in the latter half of the breeding season and 0.05 months later for each centimeter of rain above average (temperature residuals July-Nov: estimate = 0.38, SE = 0.13, t = 2.84, *P* = 0.001; rainfall residuals July-Nov: estimate = 0.05, SE = 0.03, t = 1.83, *P* = 0.07). In general, mares gave birth 0.05 months earlier for every year of age (estimate = −0.05, SE = 0.02, t = −2.03, *P* = 0.05).

**Figure 2:**
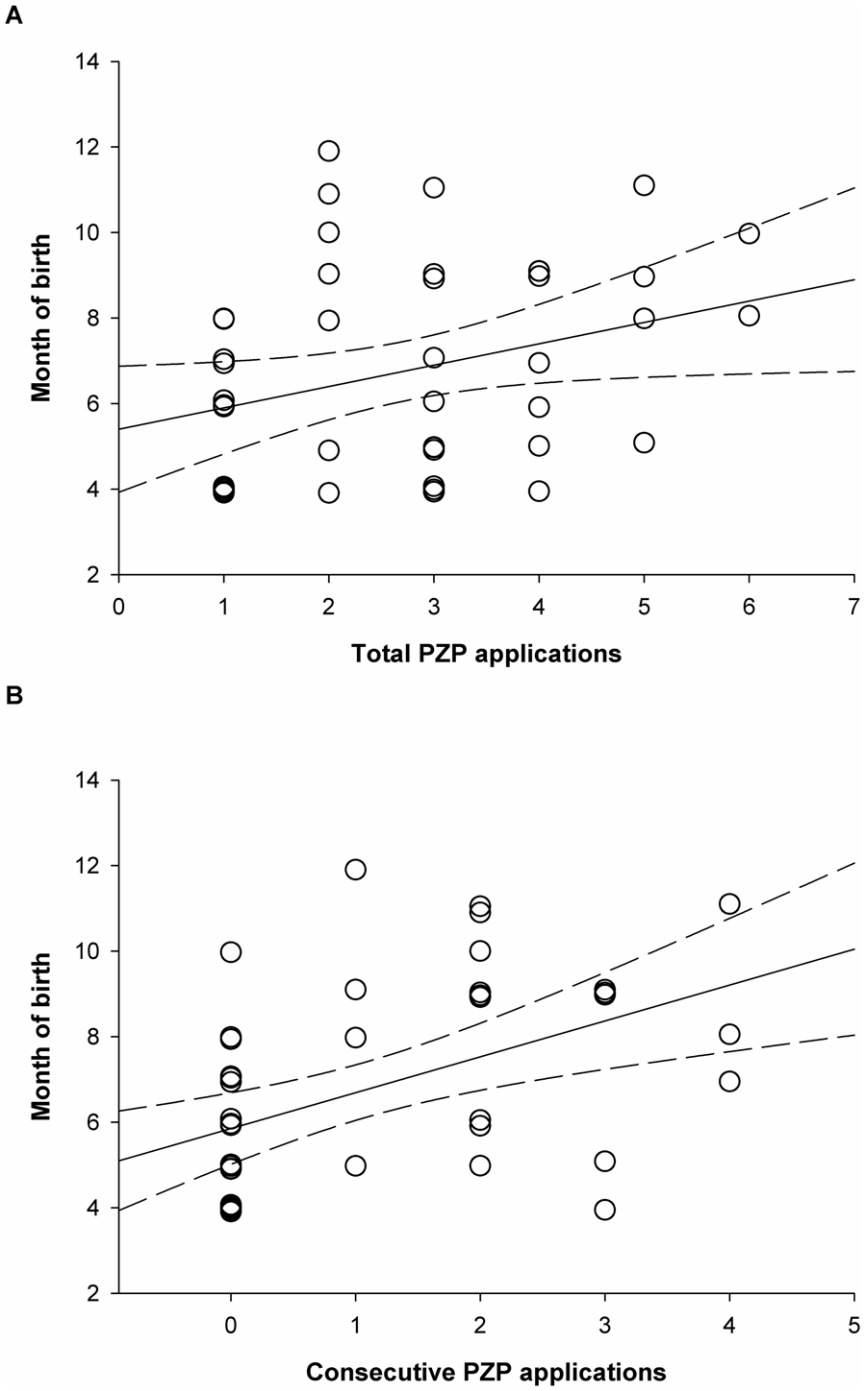
Birth month and number of A) total PZP applications, and B) consecutive PZP applications. In the events of ties, month of birth has been jittered by 0.2 years to allow clear visualization of every individual. Mares receiving more applications of PZP foaled later in the year on average than did mares receiving fewer applications. Although the number of total and consecutive applications is highly correlated, AICc suggests that the number of consecutive applications explains more of the variation in the data.

Among mares treated with PZP, those receiving a higher total number of applications foaled later in the season than did those receiving fewer applications, with each additional application associated with a 0.5 month delay in foaling date (Linear Mixed Effects Model: estimate = 0.55, SE = 0.21, t = 2.61, r^2^ = 0.65, *P* = 0.01, see Fig. 2A). In addition, each consecutive PZP application was associated with a 0.8 month delay in foaling date, on average (Linear Mixed Effects Model: estimate = 0.83, SE = 0.23, t = 3.64, r^2^ = 0.65, *P*<0.0008, see Fig. 2B). While the consecutive and total number of PZP applications were highly correlated, comparing the AICc between the prior two models suggested that the number of consecutive PZP treatments more accurately predicted month of birth than did the total number of PZP treatments (AICc total = 202.96, AICc consecutive = 196.83). The number of years that mares did not conceive (total or consecutive), however, did not correlate with foaling date (Linear Mixed Effects Model: total years: estimate = 0.31, SE = 0.26, t = 1.19, r^2^ = 0.57, *P* = 0.24; consecutive years: estimate = 0.26, SE = 0.28, t = 0.92, r^2^ = 0.44, *P* = 0.36). It is unlikely that these patterns resulted from a general trend across years, as birth dates among non-recipient animals did not change with calendar year (Linear Mixed Effects Model: r^2^ = 0.17, estimate = 0.05, SE = 0.09, t = 0.57, *P* = 0.57).

### Mare Condition

A generalized linear model, which controlled for mare age, showed that among post-contraception animals, recipient mares (both current and prior) were in better physical condition, with rump scores approximately 0.4 points higher than those of non-recipients (overall model (compared to null model): Chi Square = 7.74, *P* = 0.05; PZP treatment: estimate = 0.38, SE = 0.15, Chi Square = 6.13, *P* = 0.01; age: estimate = −0.005, SE = 0.06, Chi Square = 0.007, *P* = 0.93; PZP treatment × age: estimate = −0.02, SE = 0.06, Chi Square = 0.13, *P* = 0.72).

### Weather

**Figure 3:**
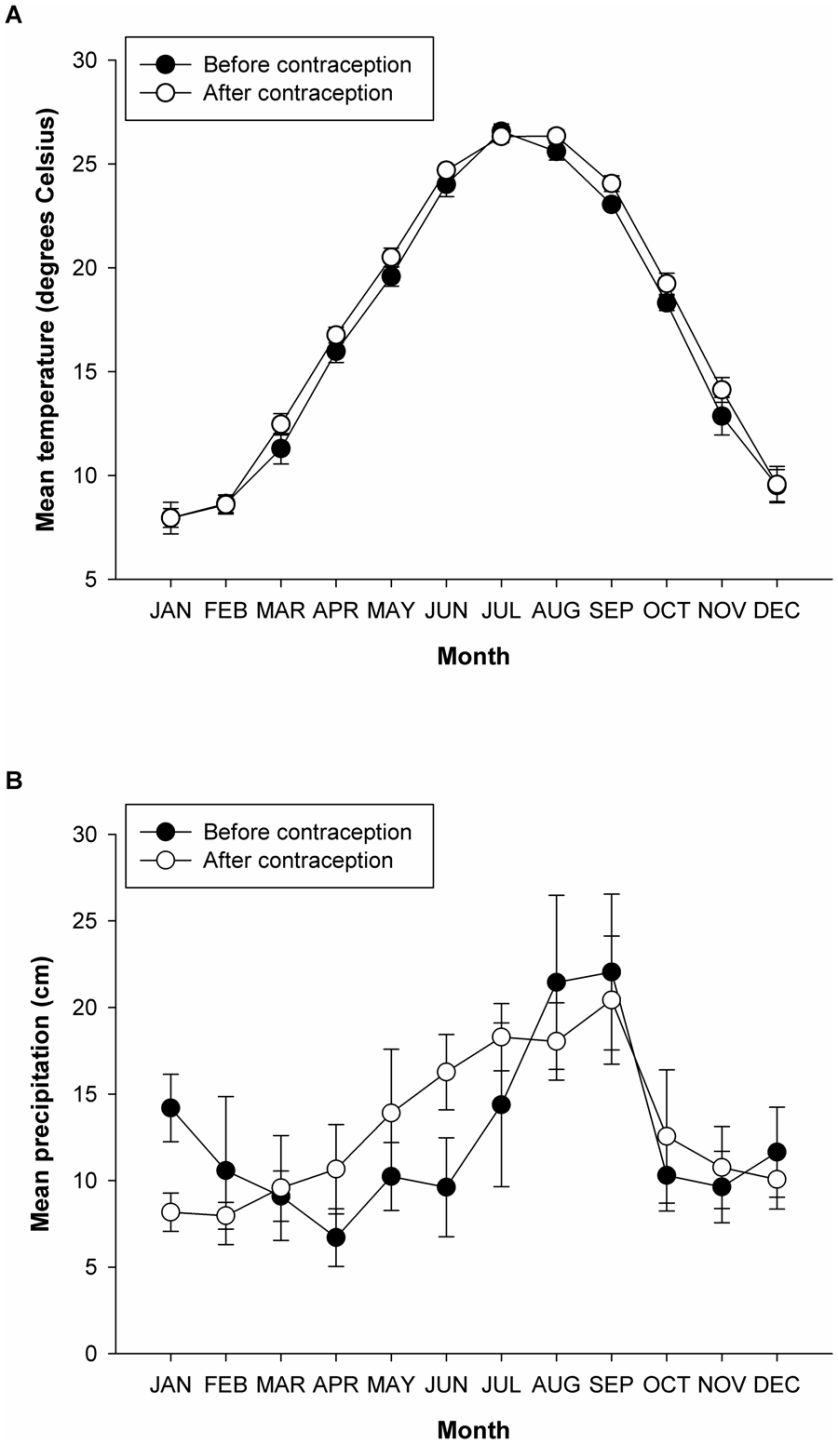
Weather data for six years pre-contraception (1995–2000) and eight years post-contraception (2001–2008) management. Data were collected from Morehead City, North Carolina, approximately 8 km from the study site (Shackleford Banks, North Carolina). Temperatures (A) were marginally warmer post-contraception than they had been pre-contraception. Overall rainfall (B) did not differ before and after contraception, though the seasonal patterns were marginally different pre- and post-contraception.

The best fit model of monthly mean temperature showed that overall temperatures were approximately 0.6°Cwarmer after the onset of contraception management (see Fig. 3A; overall model: Likelihood ratio (compared to null model)  = 358.40, *P*<0.0001, generalized r^2^ = 0.88; month: estimate = −8.49, SE = −.97, t = −8.78, *P*<0.0001; month^2^: estimate = 3.88, SE = 0.29, t = 13.43, *P*<0.0001; month^3^: estimate = −0.45, SE = 0.03, t = −13.70, *P*<0.0001; month^4^: estimate = 0.015, SE = 0.001, t = 12.20, *P*<0.0001; contraception management (before): estimate = 0.58, SE = 0.27, t = −2.13, *P* = 0.06). The best fit model of monthly precipitation showed no overall differences between the periods before and after contraception management, but there were significant interactions between month of the year and the periods before and after contraception (see Fig. 3B; overall model: Likelihood ratio (compared to null model)  = 38.43, *P*<0.0001, generalized r^2^ = 0.20, *P*<0.0001; month: estimate = −11.19, SE = 4.96, t = −2.25, *P* = 0.03; month^2^: estimate = 3.66, SE = 1.46, t = 2.51, *P* = 0.01; month^3^: estimate = −0.38, SE = 0.17, t = −2.27, *P* = 0.02; month^4^: estimate = −0.01, SE = 0.006, t = 1.88, *P* = 0.06; contraception management (before): estimate = 6.99, SE = 4.22, t = 1.66, *P* = 0.12; month × contraception management (before): estimate = −2.97, SE = 1.43, t = −2.07, *P* = 0.04; month^2^ × contraception management (before): estimate = 0.22, SE = 0.11, t = −2.03, *P* = 0.04).

### PZP Efficacy

**Figure 4:**
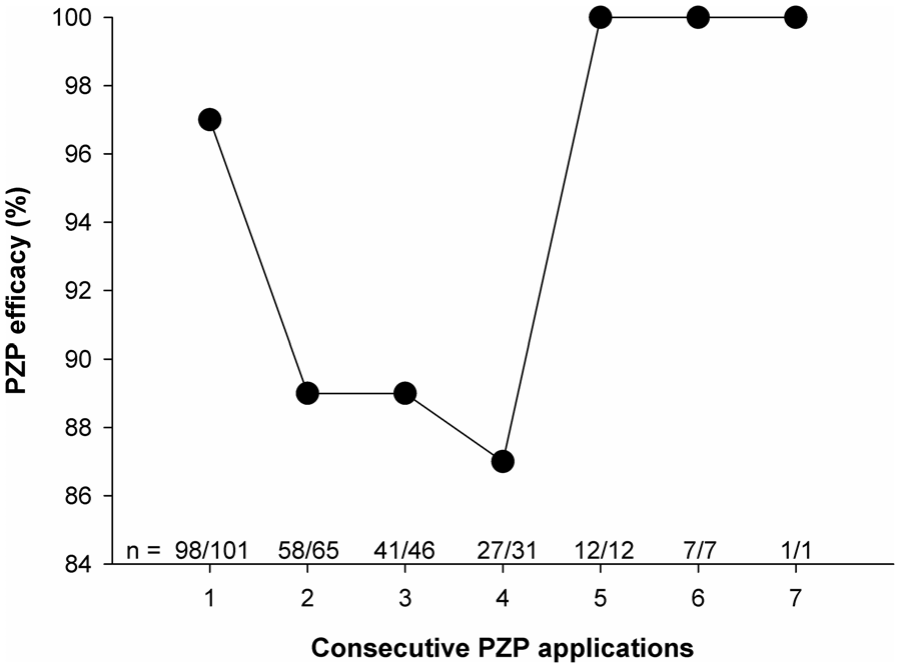
PZP efficacy and number of consecutive PZP applications. PZP efficacy was defined as the number of recipient mares that did not become pregnant divided by the total number of mares receiving the vaccine. Across the first four consecutive applications, PZP efficacy declined, returning to 100% after five or more consecutive applications (5–7 applications have been shown to result in ovulation failure and decreased oestrogen levels [5], [40]).

We defined PZP efficacy during the year of administration as the number of vaccinated mares that did not became pregnant divided by the total number receiving the vaccine. Across the first four consecutive PZP applications, this efficacy declined from 97% to 87%, returning to 100% after five or more consecutive applications (see Fig. 4). A generalized mixed effects model shows that this pattern is significant, even when controlling for mare age (overall model with binomial error distribution: Log Likelihood = −61.79, *P* = 0.01, generalized r^2^ = 0.17; consecutive PZP applications: estimate = 2.98, SE = 1.20, z = 2.49, *P* = 0.01; (consecutive PZP applications)^2^: estimate = −0.51, SE = 0.22, z = −2.33, *P* = 0.02; age at first PZP application: estimate = 0.10, SE = 0.05, z = 1.84, *P* = 0.07). Prior research has shown that five to seven years of consecutive PZP treatment can be associated with ovulation failure [4]. The present dataset is consistent with this result, as no mare receiving the vaccine for five or more consecutive years became pregnant.

## Discussion

Here we show that PZP recipients exhibited a change in their reproductive schedule: recipient mares gave birth over a broader time period than did non-recipients, with current recipients giving birth later in the year than prior recipient and non-recipient mares. Given that gestation in wild horses lasts approximately 11 to 12 months [30], this change indicates a corresponding change in the schedule of ovulatory cycling. Contraception with porcine zona pellucida is popular amongst managers specifically because it effectively reduces the odds of conception without the application of exogenous steroids [2]. Long-term studies on Assateague Island have reported that PZP has little to no effect on reproductive hormone levels, the schedule of reproductive cycling, or the social behaviors of recipient animals [4]. However, studies in other wild horse populations have shown that recipient mares both initiate and receive more instances of reproductive behavior during both the breeding [39] and non-breeding seasons [9]. This study provides the first evidence that mares treated with PZP can extend ovulatory cycling beyond the normal breeding season. This suggests that populations of wild ungulates can vary in their response to similar contraceptive treatment. Careful consideration of baseline population dynamics should be made prior to treatment in order to fully assess possible PZP effects.

### Foaling Date

Mares receiving PZP at any point during their lifetime gave birth over a broader time period than did non-recipient animals. This larger variance among PZP mares is likely driven by the fact that current recipients gave birth later than did prior recipients (see Results, Fig. 1). Moreover, mares receiving more consecutive applications foaled later in the season than did mares receiving fewer applications. Increases in the average interbirth interval for recipient mares did not seem to be driving this result, as foaling date was not affected by the number of years (cumulative or consecutive) that mares failed to conceive. This discrepancy may be due to high variability in the conception and foaling dates of treated mares. First, it is less likely that an animal vaccinated with PZP will conceive at all, thus reducing sample size. Second, due to contraceptive failure, some treated mares will conceive during the normal breeding season, further increasing variability. Interestingly, prior to the application of PZP, the average month of birth did tend to increase with interbirth interval (Linear Mixed Effects Model: estimate = 0.30, SE = 0.17, t = 1.82, r^2^ = 0.06, *P* = 0.07) [22], demonstrating at least some plasticity in the scheduling of reproductive cycling in Shackleford mares. On Assateague, PZP recipients experience normal reproductive cycling and mate at rates similar to non-recipients [40]. However, when such behavior fails to result in conception over several years, it follows that individuals extending reproductive cycling will be able to achieve conception later in the year if the contraceptive effects of PZP have decreased sufficiently [28], [29].

Because feral horses are highly social, such changes can have cascading effects on other group members and throughout the population. Our research has shown that after contraception management, PZP recipients both attract and initiate more instances of reproductive behavior [9] and are more often the harem male's nearest neighbor during the fall/winter (Nuñez, unpublished data), indicating that group spreads are reduced. Such changes represent an increase in energy expenditure and a potential decrease in nutrient intake during a time of year when sufficient energy reserves are at a premium [27]. Moreover, early foal development in unmanaged populations typically occurs during the spring and summer when resources are plentiful [11], [27]. Offspring born in the fall/winter months face nutritional and thermoregulatory challenges not experienced by their counterparts born during the normal foaling season, potentially making developmental benchmarks difficult to achieve [27].

Such predictions are not consistent with data from Assateague Island where mares show increased survival, only minimal physiological side effects, and no behavioral or demographic changes [4], [5], [6]. In addition, foal survival does not differ between foals born in or out of the normal foaling season [41]. However, on Shackleford Banks, recipient mares change groups more often, elicit and receive more instances of reproductive behavior, and receive more harassment from harem males [9], [42]. Given these differences in mare response to PZP management in the two populations, it follows that predictions based on the data from one site are not necessarily applicable to the other.

These population differences may be due to the scheduling of PZP administration at the two sites. When the contraception program on Assateague began in 1994, the priorities for treatment followed a hierarchical approach based on the previous breeding success of the population, ensuring that all mares were given an opportunity to reproduce [3]. Females for which there was a high priority for treatment included those that had produced at least one surviving offspring. Low priority females included those that were less than four years of age. Females greater than four years old that had not produced surviving offspring did not receive treatment. In addition, the plan stipulated that only mares that had produced at least three surviving offspring or two generations of offspring would receive more than three consecutive years of treatment. Foals were not to be removed as removal increases a mare's reproductive success in the subsequent year [43], [44], [45]. Finally, it was recognized that this plan was subject to change as the population numbers decreased [46]. In the present study, Shackleford mares were contracepted between 1.5 and 2 years of age and received an average of 3.4±0.2 (mean ± standard error) consecutive years of contraception, regardless of their productivity. To further control population numbers, foals born to these mares (due to contraception failure or changes in the application schedule), were likely to be removed. This difference in PZP administration and subsequent discrepancy in early life experience may contribute to the behavioral differences between the populations, as the ability to conceive with a harem male is likely critical to establishing lasting harem fidelity [16] and the retention of foals (until at least two years of age) is important to maintaining normal reproductive function [43], [44], [45].

### Possible Mechanisms

Although the effect was more pronounced in recipients of PZP, both recipients and non-recipients showed a wider range of foaling dates after contraception management (after 2001). While relatively rare, such extended periods of estrous have been documented in several equine species. Tropical species, for example, have been observed to reproduce throughout the year [12], [13], [47]. Similarly, studies of temperate species have shown that individuals can vary significantly in reproductive timing [14] and estrous behaviors during the non-breeding season [15]. Our data show that Shackleford mares exhibit at least some plasticity in their reproductive cycling. This plasticity enables mares to time their reproductive cycling according to ecological, sociological, and physiological cues.

For example, our results show that the reproductive changes exhibited by Shackleford mares correlate with warmer temperatures occurring later in the calendar year, after contraception management. Increases in rainfall late in the breeding season also correlate (albeit weakly) with later births. Both warmer temperatures and increased rainfall could result in higher resource availability [27] and afford females the additional reserves necessary to extend reproductive cycling into what is typically the non-breeding season.

The physical condition of mares may also play an important role in the extension of reproductive cycling. On Shackleford Banks, recipient mares are currently in better physical condition than are non-recipients. This is likely due to the fact that successfully contracepted mares are unconstrained by the costs of pregnancy and lactation [48]. Recipient mares will therefore have more resources to allocate to additional reproductive cycles. This effect of PZP, coupled with warmer temperatures occurring later in the year, may act to increase a mare's chances of conceiving later in the calendar year, if PZP antibody titers are sufficiently low [29].

Additionally, extended cycling in non-recipient mares could be influenced by the physiology and behavior of recipients. Shackleford males exhibit higher rates of sexual behavior towards recipient females during both breeding and non-breeding seasons [9], [42]. These overt social stimuli may entrain some non-recipients to continue reproductive behaviors and cycling into the early fall. Such stimuli are commonly used to induce receptivity in several domestic species including horses [49], pigs [50], and cows [51]. In the wild, courtship signals from conspecifics advance gonadal cycles or maturation in several taxa, including mammals [52], [53], [54], birds [55], amphibians [56], and reptiles [57]. Given the importance of social cues in the timing of reproduction among such diverse species, this possibility warrants further investigation in Shackleford mares.

Finally, the declining efficacy of PZP with increased consecutive applications is likely a contributing factor to the later foaling dates of recipient mares. Lyda and colleagues' research with captive, wild mares has shown that antibody titers against PZP remain high for up to ten months after initial treatment [28]. In addition, research with both Shackleford and Assateague horses has shown that initial applications of PZP are often effective over multiple years [9], [58], suggesting that antibody titers can remain high for longer. However, laboratory research has shown considerable variability in anti-PZP titers [29], as did Lyda and colleagues' work in which half the mares treated with PZP and Freund's Complete Adjuvant fell below contraceptive levels within the ten months of study [28]. Our data show that increasing the number of consecutive applications can reduce the single year efficacy of PZP by roughly 10%, indicating that either antibody titer or reactivity can decrease more rapidly with consecutive applications. Such patterns could result from the induction of immunological tolerance [59], which reduces responsiveness to self-tissues or repeatedly encountered, non-pathogenic antigens [60]. PZP is designed to mimic host tissue and induce an immune response against self tissue: the recipient's own zona pellucida [2]. As such, it seems reasonable that at least some animals would mount tolerance mechanisms to combat this autoimmunity. In addition, the repeated application of a specific antigen generates an antibody response that is increasingly more specific to that particular antigen [29]. The antibodies produced by mares against porcine zona pellucida should, therefore, become less cross reactive with horse zona pellucida over time. Of course, PZP efficacy will vary depending on mare age and timing of inoculation [61]. Regardless, if PZP recipients extend reproductive cycling and behavior into the non-breeding season, any decrease in efficacy that leaves them fertile in the fall/winter will help drive increases in late season conception.

Although the removal of offspring can induce estrous cycling in ungulate species [62], it is unlikely that the removal of foals has influenced foaling date among PZP-treated mares on Shackleford Banks. Thirty-nine foals (conceived due to contraception failure or administration scheduling) have been removed from the island. Approximately 55% of these foals were born to non-recipient animals. The majority of foal removals were conducted in the January following foal births. Given that non-recipient animals did not give birth later than September and most recipient animals gave birth before December, it is unlikely that foal removals in January induced late-season estrus in Shackleford mares. It is equally unlikely that increases in mare condition due to the alleviation of lactation costs resulted in early resumption of estrus the following spring [27]. If that were the case, during the early spring months we would expect to see an increase in the number of foals born to mares subjected to offspring removal. This is not borne out by the data. Still, the removal of foals is ill-advised as it increases mare fecundity the following year [43], [44], [45].

### Management Implications

When the alternative (gather and removal) is considered, PZP is currently managers' most humane and effective option for population control. However, careful study of the animals' demography, physiology, and behavior is necessary prior to and during treatment to ensure that a) the potential effects of PZP can be assessed accurately, and b) within managerial constraints, unintended effects of PZP are ameliorated. Differences in habitat, resource availability, and demography among conspecific populations will undoubtedly affect their physiological and behavioral responses to PZP contraception, and need to be considered. For instance, while Assateague horses show no behavioral and only minor physiological responses to PZP, horses on Shackleford Banks [9], [42] and in the western United States [39] alter social and reproductive behaviors in response to PZP. Our data suggest that mare condition and warming trends may present additional complications. Increases in physical condition and changes in average temperature may interact with management regimes, enabling mares to alter their reproductive physiology even further. Moreover, these data emphasize the importance of study during both the breeding and non-breeding seasons. Much of the research showing little to no effect of PZP on feral horse behavior or physiology has been performed exclusively during the breeding season [4], [5], [10], potentially missing important differences in recipient response.

If population numbers are managers' primary concern, our data show that giving five or more consecutive applications of PZP will result in 100% contraception efficacy. This is consistent with data from Assateague where mares receiving 5–7 consecutive PZP applications exhibited ovulation failure and decreased urinary oestrogen concentrations [5], [40]. However, if managers are tasked with the maintenance of natural behaviors and foaling schedules, consecutive PZP applications should be avoided. Research has shown that one application of PZP is often effective over multiple years, exhibiting yearly efficacy declines similar to that of 2–4 consecutive treatments (on Shackleford) [9], [58]. Our data show that current recipients gave birth later than both prior recipient and non-recipient animals. However, prior recipients of PZP gave birth on schedules similar to non-recipients, suggesting that breaks between treatments can ameliorate unintended behavioral and physiological changes in recipient animals. Contraception on such schedules will still maintain lower pregnancy rates, but will allow for the birth of a manageable number of offspring which are also important to the maintenance of normal behaviors [9]. These foals should be allowed to remain in the population for at least two years as earlier removal has been shown to increase a mare's reproductive success in the subsequent year [43], [44], [45]. Additionally, subadult, dispersing females should be allowed to settle into harems and have at least one foal before receiving contraception [16]. Management regimes such as this would of course necessitate a higher minimum population level. Additional research is needed to determine whether these larger, but still limited population sizes could achieve management goals. If so, this could prove a cost-effective means of controlling animal numbers while maintaining their natural physiology and behavior.

The broader implications of this research are considerable. As this study suggests, the physiological and behavioral effects of PZP are not fully understood. Still, the vaccine is currently administered to many different species including white-tailed deer, *Odocoileus virginianus* [7], elk, *Cervus elaphus* [8], black bears *Ursus americanus* [63], and African elephants, *Loxodonta Africana* [64]. As with conspecific equid populations, habitat, resource, and demographic differences among species will affect their responses to PZP contraception and need to be considered. For social species like the horse, a proper balance between managing population size and maintaining a more natural physiological and behavioral regime is particularly important.
